# Student Perceptions of Age and Ageing—An Evaluation of Swiss Dental Students Receiving Education in Gerodontology

**DOI:** 10.3390/ijerph19127480

**Published:** 2022-06-18

**Authors:** Ina Nitschke, Ulf Gegner, Werner Hopfenmüller, Bernhard A. J. Sobotta, Julia Jockusch

**Affiliations:** 1 Clinic of General, Special Care and Geriatric Dentistry, Center of Dental Medicine, University of Zurich, 8032 Zurich, Switzerland; ina.nitschke@zzm.uzh.ch (I.N.); ulf.gegner@uzh.ch (U.G.); 2 Gerodontology Section, Department of Prosthodontics and Materials Science, University of Leipzig, 04103 Leipzig, Germany; bernhard.sobotta@medizin.uni-leipzig.de; 3 Institute of Biometry and Clinical Epidemiology, Charité—Universitätsmedizin Berlin, Corporate Member of Freie Universität Berlin, Humboldt-Universität zu Berlin, and Berlin Institute of Health, 10117 Berlin, Germany; werner.hopfenmueller@charite.de; 4 University Research Priority Program “Dynamics of Healthy Aging”, University of Zurich, 8050 Zurich, Switzerland

**Keywords:** age image, aging semantic differential, Friedan’s phenomenon, old age, student perceptions

## Abstract

Society is ageing and the higher number of senior citizens in the total population is a challenge for society and often perceived as a burden. Negative images of old age can lead to ageism and poorer healthcare for older people. The younger generation will have to master these demographic challenges. Therefore, their attitude towards and their perception of the older generation has to be monitored. The aim of this study is to present the images of ageing held by dental students who received education in gerodontology and to assess possible changes between different generations of students over time and separated by gender. An annual, anonymous questionnaire survey was conducted among dental students at the end of the 10th semester each year between 2008 and 2021. The questionnaire surveyed personal attitudes towards ageing, the assessment of seniors, and personal experience with seniors (images of ageing, “Aging Semantic Differential”). In addition to confirming Friedan’s phenomenon regarding the assessment of age limits, the present study was able to demonstrate a positive image of ageing in dental students, which has remained almost constant over the years. An education in gerodontology might positively influence student perceptions of age and aging.

## 1. Introduction

Better education, modern medical care, good social conditions and, access to continuous nutrition have led to an increase in life expectancy. Höpflinger et al. described a double demographic development in highly developed countries, where, on the one hand, there is a decline in birth rates, and on the other hand, life expectancy is steadily increasing [1]. This development can also currently be observed in Switzerland [2], resulting in new challenges for society in areas such as nursing, medical care (geriatrics), and dental care (gerodontology).

At the same time, older people are often perceived as a burden on society due to their dependence [3]. In addition, it is possible that consolidated, negative images of old age may lead to ageism, which affects behaviors [4]. Relationships between the negative perceptions of old age in nurses and physicians and reduced patient involvement in treatment and care [5] or a poorer self-rated healthcare experienced by patients [6] are described in the literature. 

The younger generation will have to master these demographic challenges. Therefore, their attitude towards and their perception of the older generation and its integration into society should be continually examined and described in the form of their images of old age. Any deterioration in the younger generations’ images of old age might be seen as an indicator of social strain and possible loss of social cohesion.

The totality of ideas and attitudes towards ageing (the process of growing older) is defined in the social sciences, especially the science of ageing (gerontology), as an age image. Views on health and diseases in old age are also part of age images, as are ideas about freedom and dependence due to age-related limitations, fears and anxieties in financial and psychological areas, and thoughts about dying and death [7,8].

A distinction is made between individual and social images of old age. The individual image of age develops in the course of a person’s life through the acquisition of knowledge and understanding of age (the state of being old), one’s own expectations regarding ageing, and experiences with old people [9]. Reductions in ageism by developing educational interventions or concepts may be a possibility for altering negative age images [4,10].

Gerodontology is described as a dental field that accompanies and cares for all of the dental problems and issues related to ageing in seniors [11]. Considering the respective life situation and general condition of the older population requires interdisciplinary and multidisciplinary cooperation with other dental as well as medical specialties. Gerodontology is taught, to varying degrees, in undergraduate courses in Europe [12,13,14,15] and worldwide [16,17,18,19,20,21]. Guidelines and recommendations for teaching gerodontology were formulated by the European College of Gerodontology (ECG) [22] in 2009 and endorsed by the Association for Dental Education (ADEE) [12]. The ECG proposes the introduction of an independent department of gerodontology, the implementation of an interdisciplinary course, and the supervision of practical courses in gerodontology by dentists specialized in the field. Furthermore, the teaching of gerodontological topics both theoretically and practically and the creation of an interdisciplinary lecture series in which speakers from different disciplines are included are recommended. In addition, the integration of a case-oriented teaching course, the discussion of patient cases in different stages of functional limitation (fit/frail/in need of care), as well as practical courses, which should start after the completion of the basic theoretical teaching, should expose students to dental care for older patients within the framework of practical courses with the aim of learning how to solve simple clinical gerodontological problems [23].

Gerodontology is included in the Catalogue of Learning Objectives “Dentistry Switzerland” and is part of dental training and the state examination in Switzerland [13,24].

In the context of the Swiss Catalogue of Learning Objectives in Dentistry, it is therefore interesting to find out which age images prevail among dental students who receive gerodontological education and who will have to master the demographic challenges posed by an increasingly older patient clientele in their future professional lives. Furthermore, it is important to evaluate whether demographic change in society also causes changes in the views of students over the generations (the longitudinal perspective). To date, no data on the possible influence of a gerodontological education on dental student perceptions of age and ageing exists. Studies have shown that a specific education [4], e.g., gerontic nursing [25], or longer work experience [26] has a positive effect on attitudes towards age and ageing. Close contact with older people and more knowledge of ageism may induce positive attitudes [27].

The aim of this study is to present age images of dental students who receive gerodontological education and assess possible changes between different generations of students over time and separated by gender.

## 2. Materials and Methods

### 2.1. Study Design

The study is a cross-sectional study of dental student perceptions of age and ageing, analyzing data from a survey repeated on a yearly basis for more than 10 years. Nevertheless, it does not involve longitudinal evaluation or follow-up of students.

To ensure the quality of the data, the surveys were conducted at the same time each academic year (end of the 10th semester) assuming the same level of student knowledge and using the same questionnaire. 

### 2.2. Study Population

As part of the gerodontological training within the dental studies at the University of Zurich, Switzerland, a total of 477 dental students were surveyed about their images of age between the years 2008–2021 (*n* = 477 is the total number of students calculated from all participating students per observed year). Only dental students at the University of Zurich, Switzerland at the end of the 10th semester (each year after the same lecture ≙ the same level of education every year) were included in the study. Every year, all students of one academic year were asked to participate in the study. All students had sufficient knowledge of the German language as this is a prerequisite for obtaining a place at the university. All of them had received education in gerodontology. No inclusion or exclusion criteria regarding age or gender were applied. Similarly, it was irrelevant what previous experiences with older people the students had had outside of their studies (e.g., grandparents present or not, work in the care sector, etc.).

Only dental students were surveyed, as targeted training in the field of senior dentistry has been established for them in Switzerland [28]. Students of other disciplines, such as medicine, have the subject of geriatrics firmly anchored in their Catalogue of Learning Objectives and encounter an older and general medical patient clientele in their practical training.

### 2.3. Measuring Tools

The questionnaire used was designed in 2004 at the University of Leipzig and has been used there ever since [29,30]. In 2008, the Leipzig evaluation questionnaire was adapted to the conditions of gerodontology teaching in Zurich, Switzerland. First, evaluations over a longer period of time took place in 2014 and 2017 [23,30]. Data were evaluated every year at the same time (end of 10th semester, end of same lecture in the curriculum). The evaluation of the years 2008–2021 have been included in this analysis. The authors decided to evaluate a period of 13 years ending 2021 to minimize possible effects of the COVID-19 pandemic on the results compared to the evaluations published previously [23,30]. During COVID-19 pandemic, older people were blamed for the occurring restrictions [31], which might have had an impact on age images.

The survey was conducted anonymously and deals with personal attitudes towards ageing, the assessment of seniors, and personal experience with seniors (images of ageing).

Within the framework of the questionnaire on images of old age, demographic information for the students (age, gender) as well as their experience with people in need of care and care institutions was collected. 

Student attitudes towards the ageing process were examined with the use of half-open questions (“A woman is old from the age of…” [in years], “A man is old from the age of…” [in years], “A woman is young up to the age of…” [in years], “A man is young up to the age of…” [in years]), with the possibility of specifying age limits in absolute numbers, according to existing measurement instrument by Roux et al. (1994) [32].

The students’ own fears and hopes in the face of ageing were investigated by means of 10 given negative and positive answer options, according to Roux 1996 [33].

The recording of the student assessments of seniors was carried out with the help of the “Aging Semantic Differential” according to Rosencranz and McNevin from 1969 [34]. In three sections, the participants were given a choice of bipolar adjectives with a seven-point response scale to assess a senior. Low-value answer options indicate a positive assessment, answers with a score of four indicate a neutral assessment, and high-value answers indicate a negative assessment. In the evaluation, mean values were calculated for better comparability and illustration [9]. The first area of the “Aging Semantic Differential Scales” records the assessment of effectiveness versus ineffectiveness (“instrumental-ineffective”), the second area deals with independence versus the need for help (“autonomous-dependent”), and the third area with personal acceptance versus unacceptance (“personal acceptability-unacceptability”). 

Finally, the dynamics of generational relations were recorded using a Likert-type scale (1 = totally agree, 2 = strongly agree, 3 = rather disagree, 4 = strongly disagree) [33]. (Supplementary Materials—Description of the questionnaires).

### 2.4. Data Analysis

The sample size was not calculated since this was an explorative analysis. Similar studies have taken place with 100 participants [35,36]. Data collection was carried out with a questionnaire containing existing instruments to assess student perceptions of age and ageing as well as students’ own fears and hopes in the face of ageing [32,33,34]. 

The data collected in the years 2008–2021 were entered block-wise by semester with the program Microsoft Office Access for Windows XP 2003. Stat/Transfer Data Conversion Software Utility Version 7 was used for data transfer. Statistical analysis of the collected data was performed using SPSS [37].

The results were described for comparisons as a percentage of agreement in the respective evaluation category. Mean, median, minimum, maximum, and standard deviation were calculated for the variables observed. The Mann–Whitney U test or the Pearson Chi^2^ test were performed for comparison of independent samples where appropriate. The significance level was set at *p* < 0.05. Differences between sexes in the student attitudes towards the ageing process were calculated as delta Δ (Δ_old_ = “A man is old from the age of…” [in years]—“A woman is old from the age of…” [in years]; Δ_young_ = “A man is young up to the age of…” [in years]—“A woman is young up to the age of…” [in years]). Negative Δ values indicate that a woman is later old/longer young than a man, while positive values indicate that a man is later old/longer young than a woman. An equivalence range of at least ±5 years difference in delta Δ was considered relevant.

## 3. Results

In the years 2008–2021, a total of 477 dental students (male, 40.3%, mean age 26.6 ± 3.3 years; female, 57.4%, mean age 26.2 ± 3.0 years; no information available on sex or age of 11 students, 2.3%, mean age 23.0 ± 1.4 years) in the 10th semester participated in the survey (sample size).

Information provided by students on their experience with long-term care facilities and long-term care residents is shown in Table 1. About three-quarters of all students of both genders had already had contact with a long-term care facility at the time of the survey. Over 80% of students of both genders had contact with persons in need of long-term care. (Table 1).

### 3.1. Students Attitudes towards and Perceptions of Aging

#### 3.1.1. Attitudes towards the Ageing Process/Specification of Age Limits

Male and female student responses to the question “A man/woman is young up to the age of…” showed a significant difference (Mann–Whitney U test: *p* = 0.043), whereas no significant differences between male and female respondents were found for the other questions concerning the age limits (Table 2).

The female student Δ_old_ mean value (±SD) was 0.2 ± 3.5 years (median 0, range −10 to 20), while the male student Δ_old_ mean value (±SD) was 0.87 ± 4.3 years (median 0, range −10 to 20), indicating that male students slightly favored their own sex for being old later. 

The female student Δ_young_ mean value (±SD) was 0.48 ± 3.9 years (median 0, range −41 to 15), while the male student Δ_young_ mean value (±SD) was 1.75 ± 7.8 years (median 0, range −6 to 96), indicating that male students slightly favored their own sex for being young longer. With an equivalence range of at least ±5 years’ difference in the delta as a relevant difference, on the topic “from when is a man/woman old?”, 21.2% of the male and 25.7% of the female students evaluated this. For the topic “until when is a man/woman young?”, at least ±5 years’ difference was given by 19.0% of the male and 21.2% of the female students.

Overall, the female students estimated male gender to be young longer than their own gender. Male students preferred their own gender: a man is younger later than and longer than a woman (Friedan’s phenomenon).

#### 3.1.2. Fears and Hopes about Ageing 

The three fears most frequently mentioned by students regarding aging were “sickness” (*n* = 350, 75.6%), “loss of close relatives” (*n* = 302, 66.1%), and “physical decline” (*n* = 299, 66.0%). There were significant differences between the sexes for the fears “loss of close relatives” (Pearson Chi^2^ test, *p* = 0.035), and “social isolation” (Pearson Chi^2^ test, *p* = 0.009), with the proportion of women predominating for both fears (Table 3).

The three hopes most frequently mentioned by students regarding ageing were “time for the family” (*n* = 350, 75.8%), “cultivate friendship” (*n* = 320, 69.4%), and “tranquility/serenity” (*n* = 283, 62.2%) (multiple answers possible). Significant differences between the sexes appeared for the hopes about ageing “time for the family” (Pearson Chi^2^ test, *p* = 0.027), and ”retain my life” (Pearson Chi^2^ test, *p* = 0.048), again with the proportion of women predominating for both hopes (Table 3).

### 3.2. Aging Semantic Differentials

Semantic differential bipolar adjectives were summarized into the three dimensions “instrumental–ineffective”, “autonomous–dependent”, and “personal acceptability–unacceptability”. 

**Figure 1:**
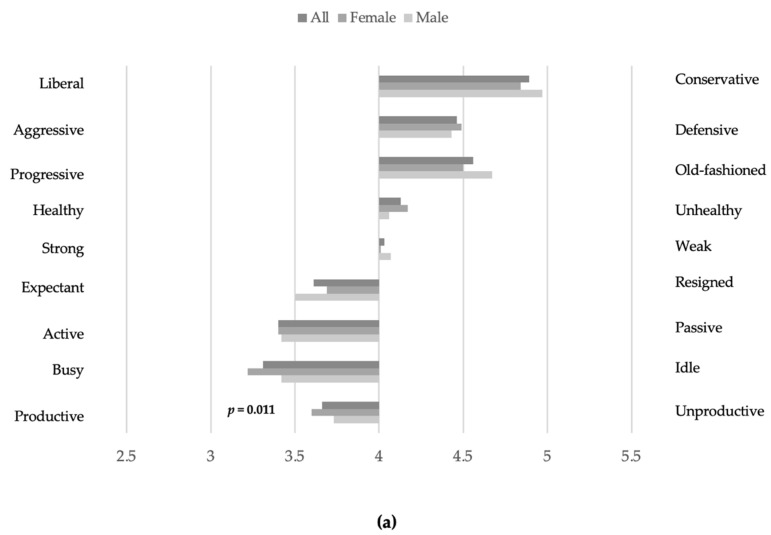

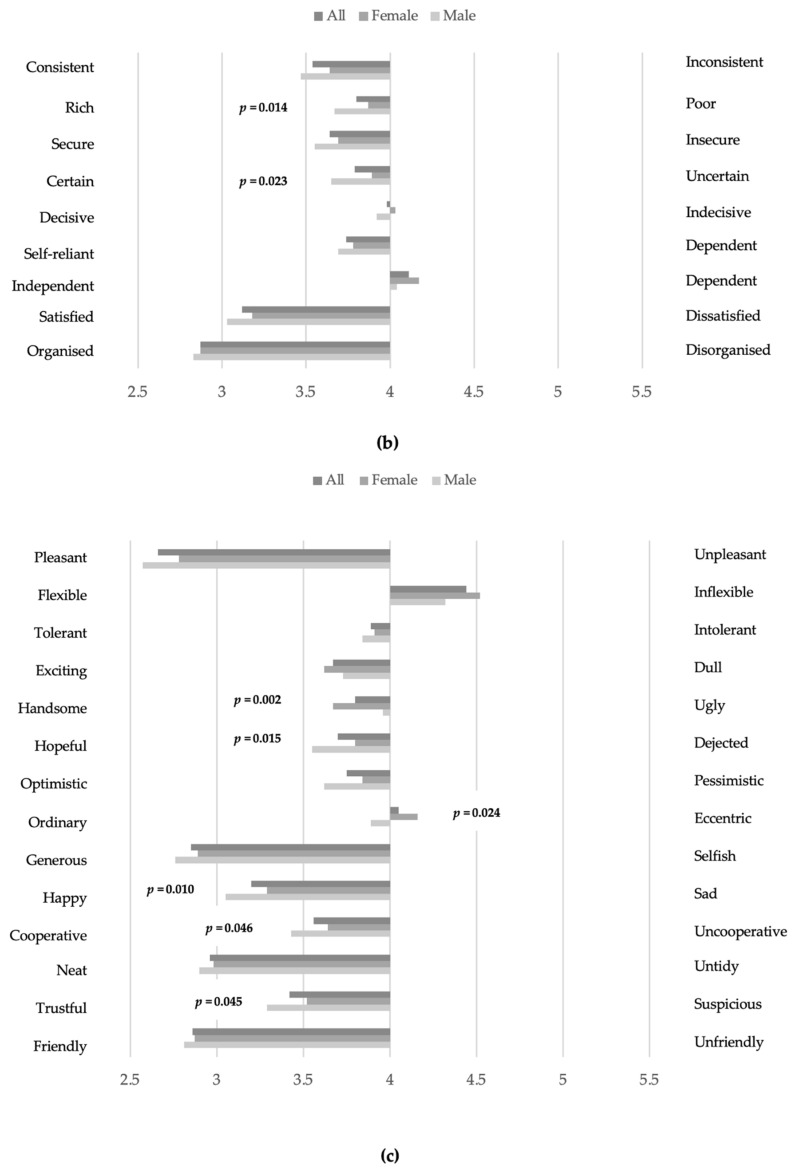
Assessment of student attitudes (mean scores, 4 = neutral attitude, the lower the score the more positive the attitude) towards seniors in the three dimensions “instrumental–ineffective”, “autonomous–dependent”, and “personal acceptability–unacceptability”. (Mann–Whitney U test, *p* ≤ 0.05). (**a**) instrumental–ineffective; (**b**) autonomous–dependent; (**c**) personal acceptability–unacceptability.

Overall, nine significant differences in mean scores by gender were observed (Mann–Whitney U test). Most of them (*n* = 6) belonged to the “personal acceptability–unacceptability” category. In most cases of significant gender differences (seven out of nine), male respondents showed a more positive attitude than female respondents by attributing lower scores (Figure 1a–c).

Combined mean scores decreased to more positive values from the “instrumental–ineffective” dimension (combined mean ± SD: 4.01 ± 1.2), “autonomous–dependent” dimension (combined mean ± SD: 3.63 ± 1.1) to the “personal acceptability–unacceptability” dimension (combined mean ± SD: 3.54 ± 1.2). The overall impression of attitudes was balanced with a positive tendency (mean 3.7 ± 1.2). 

Combined mean scores by gender showed no differences for the dimension “instrumental–ineffective” (mean ± SD: male 4.03 ± 1.2; female 3.99 ± 1.2; Mann–Whitney U test, *p* = 0.385), while there were significant gender differences for the dimensions “autonomous–dependent” (mean ± SD: male 3.54 ± 1.1; female 3.68 ± 1.1; Mann–Whitney U test, *p* ≤ 0.05) and “personal acceptability–unacceptability” (mean ± SD: male 3.43 ± 1.2; female 3.59 ± 1.2; Mann–Whitney U test, *p* ≤ 0.05). Overall, for the dimensions “autonomous–dependent” and “personal acceptability–unacceptability” male respondents showed more positive attitudes than female respondents (Figure 1a–c).

### 3.3. Dynamics of Inter-Generational Relations/Assessment of the Position of Seniors in Society

**Figure 2:**
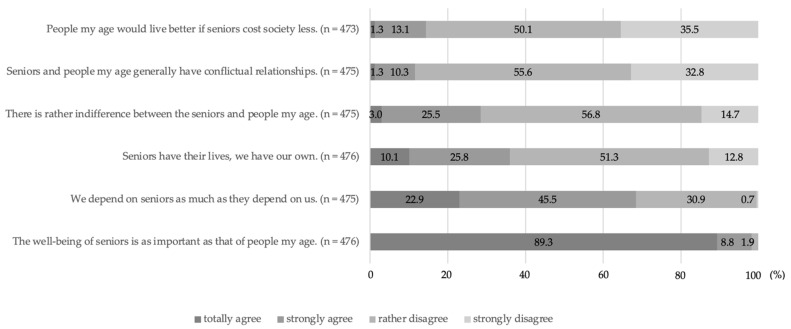
Dynamics of inter-generational relations—total student responses (multiple answers possible).

A positive dynamic of inter-generational relations was evident for all students. Negative statements about the position of seniors in society were rejected by the majority of students. Moreover, almost all students considered the well-being of seniors to be as important as their own. More than two-thirds (68.4%) of all students stated that they depend on seniors as much as seniors depend on them (Figure 2).

## 4. Discussion

### 4.1. Study Limitations

The structured evaluation form used was first applied to students at the University of Leipzig in 2008 [29] and is still part of the teaching evaluation there today. It has been used regularly to survey dental students in Zurich since 2008 and provides a data basis of 14 years. Despite minimal adaptations to the local teaching conditions in Zurich, the use of the questionnaire continues to allow comparability of data between the two universities and their teaching concepts [30].

All participating students were at the end of their gerodontological training, about to take their state examination. Thus, the study participants had completed the theoretical and practical part of their training in senior dentistry and were able to gain the insights and experience in gerodontology offered to them. While the practical training is compulsory, a guarantee of full participation in the gerodontology lecture series and the gerodontological revision course cannot be given, as these lectures were not compulsory. The results should be viewed under this aspect.

### 4.2. Student Activity in Care

These results show a constantly positive attitude of the study participants towards the work in care, which they regarded as a rather more physical than mental effort, as described by Stillhart [23]. Perrig-Chiello et al. published differing results when examining family caregivers where high psychological stress was observed, such as feeling depressed, tense, nervous and under chronic stress [38]. Reasons for this discrepancy could be the fact that the caregivers were relatives, and their care was probably provided in addition to their occupation.

Despite the positive description of the experiences in long-term care facilities, the majority of the dental students in Zurich rejected the mandatory introduction of a nursing internship. Possible reasons for this could be the already very extensive curricula of the dental school or the fact that this unpaid internship would have to take place during the lecture-free period. In Germany, the completion of a one-month nursing internship, as well as a four-week “famulatur” (internship in a dental practice), has been mandatory since the introduction of the new licensing regulations [39].

### 4.3. Attitudes towards the Ageing Process/Specification of Age Limits

Comparing the data on the age limits of students, it is noticeable that the data of students of both genders on the age limits, i.e., from when a man/woman is old or until when a man/woman is young, remains relatively constant over the years, as already described by Filipp and Mayer [40].

### 4.4. Fears and Hopes about Ageing

The presentation of fears about old age shows that fears in areas such as health and the loss of relatives are greater than financial and economic fears. This can certainly be attributed to the expectation of achieving above average incomes when working in a professional position after completion of academic training. Social aspects, such as having more time for family, friends, and for oneself, dominated among the greatest hopes. The comparison with Demmerle’s data and the data of this study shows no change. The above-mentioned fears and hopes are mentioned in the same order. Only the percentage comparison reveals a slight redistribution of fears of illness to other areas [30]. An explanation of this trend could be the steadily advancing development in the therapy of diseases in old age (e.g., dementia), as well as the objectives of policies implemented in favour of older and ageing people described by Hopflinger and Stuckelberger. Solidarity between older and younger people and maintaining of the independence of the older generations are factors which facilitate active participation of seniors in social events [41].

Berner examined images of ageing among medical students; here, too, positive (active, happy) as well as negative (sickly, inflexible) aspects were mentioned. He described ageing with changes, which can be perceived as gain and as loss. In addition, he mentions a possible influence of the negative experiences of students occurring in the health care sector in the context of their education on their attitudes towards seniors [42]. If the attitude of the medical students summarized by Berner is compared with the results of this study, this negative development of the attitude towards seniors cannot be confirmed. The students of dentistry have a very positive attitude towards seniors. Their well-being and a harmonious coexistence seem to be very important to them.

### 4.5. Aging Semantic Differential

The aging semantic differential showed that male respondents had a more positive attitude than females towards older people. This contradicts the statements of Schlegel, according to whom women grant seniors significantly greater independence and tend to focus more on the productivity of seniors than men [43]. The overall impression of attitudes in this study was balanced with a positive tendency, whereas in Schlegel’s study seniors were seen as slightly ineffective with regard to activity in everyday life but still as independent individuals who were personally accepted by the students [43].

### 4.6. Dynamics of Inter-Generational Relations/Assessment of the Position of Seniors in Society

The current assessment of dental students in Zurich with regard to seniors can be described as neutral to positive on the basis of the information provided. Compared with a previous analysis, a consistently harmonious picture of relations with the elderly is thus evident [23].

## 5. Conclusions

In addition to confirming Friedan’s phenomenon regarding the assessment of age limits, the present study was able to demonstrate a positive age image of dental students, which has remained almost constant over many years. It can be assumed that undergraduate teaching in gerodontology and the associated insights and contacts with older people may be factors supporting a positive trend in the student perceptions of age and ageing. However, such an influence on the age picture of the undergraduate training received and, if applicable, postgraduate training cannot be quantified and should be investigated in further studies. It would have to be shown whether this positive age picture persists after graduation for licensed dentists or whether there are differences between students with and without, or before and after, training in gerodontology. 

## Figures and Tables

**Table 1:** Student experience with long-term care facilities and long-term care residents. (* experience in care = means both professionally in the context of an education or learned profession or as a layperson) (different *n* as total: not all students responded to all questions).

Item	Dental Students (*n* = 477)			
	Male	Female	No Gender	Total
**Contact with long-term care facilities** (outside of the dental curriculum) (*n*/%)	*n* = 144	*n* = 238	*n* = 11	*n* = 393
No	34/23.6	61/25.6	3/27.3	98/24.9
Yes	110/76.4	177/74.4	8/72.7	295/75.1
**Contact with long-term care residents** (outside of the dental curriculum) (*n*/%)	*n* = 143	*n* = 238	*n* = 11	*n* = 392
No	26/18.2	40/16.8	4/36.4	70/17.9
Yes	117/81.8	198/83.2	7/63.6	322/82.1
Experience in care * (*n*/%)	*n* = 191	*n* = 274	*n* = 11	*n* = 476
No	72/37.7	110/40.1	6/54.5	188/39.5
Yes	119/62.3	164/59.9	5/45.5	288/60.5
**Relatives in need of care at present** (*n*/%)	*n* = 190	*n* = 273	*n* = 11	*n* = 474
No	107/56.3	178/65.2	10/90.9	295/62.2
Yes	83/43.7	95/34.8	1/9.1	179/37.8
**Involvement in the care of relatives at present** (*n*/%)	*n* = 48	*n* = 73	*n* = 1	*n* = 122
No	30/62.5	45/61.6	1/100	76/62.3
Yes	18/37.5	28/38.4	0/0	46/37.7
**Number of hours per week (present)** (hours)	*n* = 9	*n* = 14	*n* = 0	*n* = 23
Mean ± SD	1.8 ± 0.9	2.9 ± 2.7	/	2.5 ± 2.2
Median (Range)	2 (0.5–3)	1.5 (1–10)	/	2 (0.5–10)
**Relatives in need of care in the past** (*n*/%)	*n* = 189	*n* = 272	*n* = 11	*n* = 472
No	51/ 27.0	84/30.9	4/36.4	139/29.4
Yes	138/73.0	188/68.6	7/63.6	333/70.6
**Involvement in the care of relatives in the past** (*n*/%)	*n* = 115	*n* = 179	*n* = 7	*n* = 301
No	69/60.0	99/55.3	6/85.7	174/57.8
Yes	46/40.0	80/44.7	1/14.3	127/42.2
**Number of hours per week (past)** (hours)	*n* = 22	*n* = 52	*n* = 1	*n* = 74
Mean ± SD	3.1 ± 2.8	5.0 ± 5.3	3.0	4.4 ± 4.7
Median (Range)	2 (0.5–10)	4 (0.25–28)	3.0	3.0 (0.25–28)
**Work in care currently alongside the dental curriculum** (*n*/%)	*n* = 191	*n* = 271	*n* = 11	*n* = 473
No	133/69.6	221/81.5	10/90.9	364/77.0
Yes	58/30.4	50/18.5	1/9.1	109/23.0
**Number of hours per week (alongside)** (hours)	*n* = 14	*n* = 16	*n* = 1	*n* = 31
Mean ± SD	12.1 ± 25.5	6 ± 2.9	8.0	8.8 ± 17.2
Median (Range)	6 (0–100)	5.5 (0–12)	8	6 (0–100)
**Work in care before the dental curriculum** (*n*/%)	*n* = 187	*n* = 264	*n* = 10	*n* = 461
No	80/42.8	143/54.2	7/70.0	203/49.9
Yes	107/57.2	121/45.8	3/30.0	258/50.1
**Perception of the work in care** (*n*/%) (multiple answers possible)	*n* = 301	*n* = 429	*n* = 9	*n* = 739
Boring	10/3.3	7/1.6	0/0	17/2.3
Interesting	50/16.6	75/17.5	1/11.1	126/17.1
Versatile	49/16.3	66/15.4	2/22.2	117/15.8
One-sided	9/3.0	3/0.8	0/0	12/1.6
Psychologically stressful	24/8.0	40/9.3	1/11.1	65/8.8
Physically stressful	34/11.3	67/15.6	2/22.2	103/13.9
Pleasant	51/16.9	58/13.5	1/11.1	110/14.9
Compassion towards people in need of care	19/6.3	40/9.3	0/0	59/8.0
New human experience	55/18.3	73/17.0	2/22.2	130/17.6
**Would you welcome the introduction of a care internship as part of the dental curriculum?** (*n*/%)	*n* = 191	*n* = 274	*n* = 10	*n* = 470
No	115/60.2	168/62.5	9/81.8	292/62.1
Yes	76/39.8	101/37.5	1/9.1	178/37.9

**Table 2:** Student definitions of “young” and “old” by year (2008–2021) and by gender. (different n per gender and question: * *n* = 22 for female students and question “A woman is young up to the age of…”; *n* = 12 for male students and question “A woman is old from the age of…”) (bold values indicate *p* ≤ 0.05) (based on data from (23) and (30)).

			“A Woman Is Old from the Age of…” [in Years]		“A Man Is Old from the Age of…” [in Years]		“A Woman Is Young up to the Age of…” [in Years]		“A Man Is Young up to the Age of…” [in Years]	
			Male	Female	Male	Female	Male	Female	Male	Female
Year of Evaluation	*n*_students_		Mean ± SD	Mean ± SD	Mean ± SD	Mean ± SD	Mean ± SD	Mean ± SD	Mean ± SD	Mean ± SD
	Male	Female	Median (Range)	Median (Range)	Median (Range)	Median (Range)	Median (Range)	Median (Range)	Median (Range)	Median (Range)
2008	16	14	69.2 ± 9.9	68.7 ± 4.6	69.4 ± 8.1	66.6 ± 6.3	41.4 ± 16.1	43.6 ± 10.5	41.3 ± 15.6	43.9 ± 10.4
			69 (50–85)	70 (60–75)	70 (50–80)	70 (50–75)	38.5 (20–80)	40 (30–65)	40 (20–80)	40 (30–65)
2009	11	23	70.6 ± 8.3	69.4 ± 8.3	71.8 ± 6.0	69.1 ± 7.2	36.7 ± 4.6	39.5 ± 8.4	37.4 ± 4.9	40.0 ± 8.4
			70 (55–80)	70 (50–85)	70 (60–80)	70 (55–80)	40 (30–40)	40 (20–65)	40 (30–45)	40 (20–65)
2010	14	28	65.2 ± 11.0	70.0 ± 8.2	68.6 ± 6.3	70.5 ± 6.9	32.9 ± 5.8	38.8 ± 12.1	37.9 ± 3.7	39.2 ± 12.1
			65 (40–85)	70 (50–90)	70 (60–80)	70 (60–90)	32.5 (25–40)	37.5 (20–80)	40 (30–40)	37.5 (20–80)
2011	12	14	63.3 ± 10.3	69.5 ± 9.3	64.2 ± 9.0	68.3 ± 7.9	37.6 ± 7.2	37.9 ± 7.8	38.7 ± 9.1	37.4 ± 8.2
			65 (45–80)	70 (50–85)	65 (45–80)	70 (50–80)	35 (30–50)	35 (30–60)	37.5 (30–59)	35 (25–60)
2012	7	21	72.9 ± 9.1	73.0 ± 7.0	72.1 ± 7.0	72.9 ± 5.8	38.0 ± 8.3	39.8 ± 8.6	39.0 ± 8.8	41.1 ± 9.2
			75 (55–80)	75 (60–85)	70 (60–80)	75 (65–80)	40 (26–50)	40 (25–65)	40 (28–50)	40 (30–65)
2013	8	25	60.0 ± 11.7	69.3 ± 8.5	63.1 ± 7.5	69.4 ± 6.4	34.3 ± 6.1	39.1 ± 8.7	36.1 ± 6.3	39.0 ± 8.4
			65 (40–70)	70 (50–80)	65 (50–70)	70 (60–82)	35 (25–45)	35 (29–60)	37 (25–45)	35 (30–60)
2014	12	17	70.8 ± 16.6	68.2 ± 9.8	70.8 ± 16.2	68.5 ± 8.4	42.2 ± 11.6	43.0 ± 14.1	43.0 ± 11.0	43.1 ± 14.2
			75 (30–90)	70 (50–85)	75 (30–90)	70 (50–85)	37.5 (30–65)	40 (20–70)	40 (30–65)	40 (20–70)
2015	10 *	20 *	63.6 ± 17.3	61.6 ± 10.9	68.7 ± 10.7	63.5 ± 11.0	30.7 ± 7.6	36.8 ± 6.9	36.1 ± 5.3	38.0 ± 7.8
			65.5 (30–90)	65 (36–75)	67.5 (50–90)	70 (38–75)	32.5 (18–40)	35 (25–50)	35 (30–45)	35 (25–55)
2016	12	14	72.2 ± 7.3	69.4 ± 6.9	72.8 ± 5.1	69.1 ± 6.3	32–7 ± 8.7	38.0 ± 9.2	34.3 ± 9.8	38.7 ± 9.0
			73.5 (60–80)	70 (60–80)	72.5 (65–80)	70 (60–80)	30 (21–45)	35 (25–55)	30 (21–50)	37 (25–60)
2017	10	20	63.5 ± 14.4	69.6 ± 6.6	64.6 ± 13.9	69.6 ± 6.6	30.5 ± 6.4	36.5 ± 6.3	31.0 ± 7.2	37.3 ± 6.2
			69.5 (33–75)	70 (60–85)	68.5 (39–75)	70 (60–85)	29 (25–45)	35 (25–50)	29 (24–45)	37.5 (30–50)
2018	11	14	62.8 ± 17.1	69.3 ± 12.7	64.2± 16.3	70.4 ± 9.3	35.2 ± 12.2	39.9 ± 15.7	36.6 ± 11.3	42.4 ± 14.9
			65 (16–73)	70 (35–80)	70 (20–80)	70 (45–80)	35 (15–60)	36 (16–80)	35 (19–60)	40 (18–80)
2019	20	23	70.5 ± 5.7	70.8 ± 7.0	70.3 ± 4.6	70.0 ± 6.6	39.1 ± 9.2	38.8 ± 8.5	38.6 ± 9.0	39.8 ± 8.6
			70 (65–85)	75 (50–80)	70 (65–80)	70 (50–80)	36 (20–60)	40 (20–55)	36 (20–60)	40 (25–55)
2020	27	16	69.5 ± 7.4	68.8 ± 8.3	69.4 ± 6.3	70.1 ± 6.0	42.3 ± 10.6	38.4 ± 7.5	42.8 ± 10.7	38.1 ± 6.0
			70 (50–80)	70 (50–80)	70 (50–80)	70 (60–80)	45 (20–70)	35.5 (25–59)	45 (20–70)	38 (25–50)
2021	19	20	69.7 ± 6.6	68.2 ± 6.0	69.5 ± 5.5	69.9 ± 7.5	35.1 ± 8.5	35.3 ± 8.9	35.8 ± 8.2	34.3 ± 7.8
			70 (60–80)	70 (60–79)	70 (60–80)	70 (60–89)	35 (16–50)	34 (20–60)	35 (18–50)	35 (19–50)
All	189	269	67.9 ± 10.9	69.1 ± 8.5	68.8 ± 9.1	69.3 ± 7.5	37.1 ± 10.1	38.9 ± 9.7	38.3 ± 9.7	39.3 ± 9.7
			70 (16–90)	70 (35–90)	70 (20–90)	70 (38–90)	35 (15–80)	38 (16–80)	39 (18–80)	40 (18–80)
Mann–Whitney U			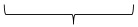		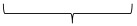		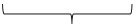		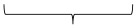	
			*p* = 0.559		*p* = 0.980		***p* = 0.043**		*p* = 0.333	

**Table 3:** Student (a) fears and (b) hopes about ageing by year, and «All» by gender. (* data not assessed in 2020/2021) (multiple answer possible) (bold values indicate *p* ≤ 0.05) (based on data from (23) and (30)).

	**(a) Fears about Ageing**																			
	**Sickness**		**Loss of Close Relatives**		**Physical Decline**		**Loss of Independence**		**Psychological Decline**		**Social Isolation**		**Reduced Activity**		**Boredom**		**Approaching Death**		**No Employment Anymore**	
**Year of Evaluation**	***n***	**%**	***n***	**%**	***n***	**%**	***n***	**%**	***n***	**%**	***n***	**%**	***n***	**%**	***n***	**%**	***n***	**%**	***n***	**%**
2008	27	84.4	19	59.5	19	59.4	21	65.6	16	51.6	10	31.2	9	28.1	3	9.4	6	18.7	1	3.1
2009	24	70.6	21	61.8	22	64.7	20	58.8	10	29.4	7	20.6	14	41.2	5	14.7	6	17.65	3	8.8
2010	39	88.4	35	83.7	28	65.1	30	69.8	12	27.9	24	55.8	14	32.6	12	27.9	6	11.63	6	13.9
2011	21	80.8	16	61.5	16	61.5	16	61.5	10	38.5	10	38.5	8	30.8	5	19.2	3	11.5	1	3.8
2012	19	65.5	19	65.5	14	48.3	22	75.9	11	37.9	5	17.2	8	27.6	5	17.2	5	17.2	1	3.4
2013	26	78.8	22	66.7	17	51.5	22	66.7	14	42.4	4	12.1	4	12.1	5	15.1	7	21.2	3	9.1
2014	23	79.3	19	65.5	19	65.5	17	58.6	9	31.0	11	37.9	9	31.0	7	24.1	6	20.7	5	17.2
2015	19	59.4	18	56.2	21	65.6	18	56.2	13	40.6	9	28.1	8	25.0	5	15.6	5	15.6	1	3.1
2016	20	71.4	16	57.1	20	71.4	12	42.9	11	39.3	11	39.3	8	28.6	7	25.0	4	14.3	6	21.4
2017	21	70.0	20	66.7	22	73.3	16	53.3	13	43.3	10	33.3	8	26.7	7	23.3	2	6.7	2	6.7
2018	17	53.1	16	50.0	20	62.5	17	53.1	9	28.1	15	46.9	13	40.6	9	28.1	5	15.6	2	6.2
2019	32	74.4	26	60.5	22	51.2	24	55.8	15	34.9	13	30.2	9	20.9	9	20.9	7	16.3	3	7.0
2020	32	74.4	34	79.1	29	67.4	26	60.5	14	32.6	16	37.2	17	39.5	7	16.3	*	*	4	9.3
2021	30	75.0	21	52.5	30	75.0	20	50.0	15	37.5	16	40.0	113	32.5	8	20.0	*	*	5	12.5
All	350	75.6	302	66.1	299	66.0	281	61.8	172	38.7	161	36.3	142	32.3	94	21.4	62	17.5	43	9.9
Out of all:																				
Male	140	40.0	111	36.8	119	39.8	105	37.4	76	44.2	50	31.1	59	41.5	41	43.6	20	32.3	19	44.2
Female	205	58.6	187	61.9	171	57.2	171	60.9	91	52.9	106	65.8	80	56.3	52	55.3	41	66.1	24	55.8
No indication of sex available	5	1.4	4	1.3	9	3.0	5	1.7	5	2.9	5	3.1	3	2.2	1	1.1	1	1.6	0	0
*p* (Pearson Chi^2^ test; male vs. female)	0.841		**0.035**		0.977		0.118		0.125		**0.009**		0.709		0.485		0.505		0.610	
	**(b) Hopes towards Ageing**																			
	**Time for the Family**		**Cultivate Friendship**		**Tranquility/Serenity**		**Time for Myself**		**Liberated from Obligations**		**Retain my Lifestyle**		**Converse**		**Not Having to Work Anymore**		**Be Available for Others**		**Start a New Life**	
**Year of Evaluation**	***n***	**%**	***n***	**%**	***n***	**%**	***n***	**%**	***n***	**%**	***n***	**%**	***n***	**%**	***n***	**%**	***n***	**%**	***n***	**%**
2008	17	54.8	21	67.7	16	51.6	19	61.3	13	43.3	10	32.3	6	19.3	8	25.8	5	16.1	6	19.3
2009	28	82.3	24	70.6	18	52.9	18	52.9	8	23.5	15	44.1	10	29.4	7	20.6	7	20.6	7	20.6
2010	33	76.7	32	74.4	29	67.4	28	65.1	16	37.2	21	48.8	12	27.9	8	18.6	12	27.9	5	11.6
2011	21	80.8	19	73.1	18	69.2	14	53.8	9	34.6	13	50.0	8	30.8	2	7.7	2	7.7	4	15.4
2012	21	72.4	23	79.3	18	62.1	19	65.5	11	37.9	17	58.6	10	34.5	4	13.8	8	27.6	5	17.2
2013	27	81.8	24	72.7	22	66.7	19	57.6	17	51.5	12	36.4	10	30.3	11	34.4	9	27.3	9	27.3
2014	25	89.3	19	67.9	16	57.1	21	75.0	13	46.4	15	53.6	12	42.9	8	28.6	8	28.6	6	21.4
2015	21	63.6	20	60.6	19	57.6	14	42.4	13	39.4	10	30.3	7	21.2	5	15.1	7	21.2	5	15.2
2016	21	75.0	20	71.4	16	57.1	14	50.0	20	71.4	9	32.1	12	42.9	7	25.0	5	17.9	7	25.0
2017	23	76.7	20	66.7	17	56.7	17	56.7	16	53.3	11	36.7	8	26.7	10	33.3	3	10.0	4	13.3
2018	20	62.5	18	56.2	19	59.4	19	59.4	14	43.7	8	25.0	8	25.0	8	25.0	3	9.4	2	6.2
2019	32	74.4	31	72.1	25	58.1	20	46.5	17	39.5	14	32.6	7	16.3	10	23.3	9	20.9	8	18.6
2020	34	79.1	26	60.5	31	72.1	24	55.8	20	46.5	17	39.5	8	18.6	10	23.3	7	16.3	7	16.3
2021	27	67.5	23	57.3	19	47.5	18	45.0	21	52.5	11	27.5	9	22.5	11	27.5	10	25.0	3	7.5
All	350	75.8	320	69.4	283	62.2	264	58.7	208	46.6	183	41.2	127	29.1	109	24.8	95	21.6	78	17.8
Out of all:																				
Male	132	37.7	121	37.8	117	41.3	107	40.5	89	42.8	84	45.9	52	40.9	50	45.9	37	38.9	28	35.9
Female	212	60.6	192	60.0	163	57.6	150	56.8	116	55.8	96	52.5	74	58.3	55	50.5	56	58.9	48	61.5
No indication of sex available	6	1.7	7	2.2	3	1.1	7	2.7	3	1.4	3	1.6	1	0.8	4	3.6	2	2.2	2	2.6
*p* (Pearson Chi^2^ test; male vs. female)	**0.027**		0.060		0.486		0.663		0.275		**0.048**		0.908		0.120		0.847		0.472	

## Data Availability

The data presented in this study are available from the corresponding author upon request. The data are not publicly available for ethical reasons.

## Supplementary Materials

The following supporting information can be downloaded at: https://www.mdpi.com/article/10.3390/ijerph19127480/s1, File S1: Description of the questionnaires.
