# Supplementary material for: Student Perceptions of Age and Ageing—An Evaluation of Swiss Dental Students Receiving Education in Gerodontology
Source: Int J Environ Res Public Health. 2022 Jun 18;19(12):7480. doi: 10.3390/ijerph19127480 (PMC9223551; doi:10.3390/ijerph19127480)
Supplement: Supplementary file 1 [file ijerph-19-07480-s001.zip › ijerph-1779300-supplementary.pdf]

# Students` Perceptions towards Age and Ageing – an evaluation of Swiss dental students receiving education in gerodontology

**Ina Nitschke, Ulf Gegner, Werner Hopfenmüller, Bernhard A.J. Sobotta, and Julia Jockusch**

## Supplementary Material - Description of the questionnaires

### Part 1 – Sociodemographic items

- Age [in years]
- Place of birth [categories: within Switzerland: yes/no]
- Sex [categories: male, female]

### Part 2 – Attitudes towards age and aging

- Attitude towards aging:
  - "A woman is old from the age of..." [in years]
  - "A man is old from the age of..." [in years]
  - «A woman is young up to the age of…" [in years]
  - «A man is young up to the age of…" [in years]
- Fears towards aging [categories: yes/no, multiple answers possible]
  - Sickness
  - Loss of close relatives
  - Physical decline
  - Loss of independence
  - Psychological decline
  - Social isolation
  - Reduced activity
  - Boredom
  - Approaching death
  - No employment anymore
- Hopes about aging [categories: yes/no, multiple answers possible]
  - Time for the family
  - Cultivate friendship
  - Tranquility/serenity
  - Time for myself
  - Liberated from obligations
  - Retain my lifestyle
  - Converse
  - Not having to work any more

  - Be available for others
  - Start a new life
- Aging Semantic Differential Scale *(«I rate seniors as…»)*
  - instrumental–ineffective
  - autonomous–dependent
  - personal acceptability-unacceptability
- Questions about seniors
  [categories Lickert-Scale: totally agree, strongly agree, rather disagree, strongly disagree]
  - The well-being of seniors is just as important as that of people my age.
  - We depend on seniors as much as they depend on us.
  - Seniors have their lives, we have our own.
  - There is rather indifference between the seniors and people my age.
  - Seniors and people my age generally have conflictual relationships.
  - People my age would live better if seniors cost society less.

**Part 3 – Questions about your studies**

- What semester are you in today? [number of semester]
- Did you work on the gerostomatological case in the POL course? [categories yes/no]
- Did you regularly attend the lecture series "Senior Dentistry" in the 9th semester?
- Did you participate in the "Geriatrics in Practice" mantle course in the 3rd or 4th year of study?

**Part 4 – Questions about your older relatives**

- Do you have any living grandmothers? [categories: no, one, two]
- How old are they? [in years]
- Do you have any living grandfathers? [categories: no, one, two]
- How old are they? [in years]
- do the grandparent(s)/grandmother(s)/grandfather(s) live in the same house/apartment of your family? [categories: yes/no]

**Part 5 – Questions about your experience in caregiving**

- Have you ever had contact with people in need of care in your life, other than in mobiDent settings? [categories: yes/no]
- Have you had any contact with nursing facilities in your life, other than in mobiDent use? [categories: yes/no]
- Have you had any experience in nursing care? [categories: yes/no]
- Is a relative of yours currently in need of care? [categories: yes/no]
  - Are you involved in providing care? [categories: yes/no]

    - How many hours a week are you involved in caregiving? [in hours per week]
- Has a relative of yours needed care? [categories: yes/no]
    - Were you involved in providing care? [categories: yes/no]
    - How many hours a week are you involved in caregiving? [in hours per week]
- Do you currently work in nursing (nursing facility/hospital) in addition to your studies? [categories: yes/no]
    - How many hours a week do you work in nursing? [in hours per week]
- Have you ever worked in a nursing facility? [categories: no; yes as an employee; yes, as an intern; yes, as a community service worker; yes, other]
- How did you find working in nursing? (multiple answers possible) [categories: boring; interesting; varied; one-sided, psychologically stressful, physically demanding, I enjoyed helping, I felt sorry for the seniors, it was a new human experience for me; other]
- Would you like to see a nursing internship introduced in dental school as a compulsory course, as it is for medical students? [categories: yes/no]
